# Breaking the Fifth Wall: Two Studies of the Effects of Observing Interpersonal Communication with Content Creators on YouTube

**DOI:** 10.3390/bs14020140

**Published:** 2024-02-16

**Authors:** Ezgi Ulusoy, Brandon Van Der Heide, Siyuan Ma, Kelsey Earle, Adam J. Mason

**Affiliations:** 1 Department of Communication, Michigan State University, East Lansing, MI 48824, USA; vdheide@msu.edu (B.V.D.H.); masonad1@msu.edu (A.J.M.); 2 Department of Communication, University of Macau, Macao 999078, China; siyuanma@umac.mo; 3 Department of Economics and Management, Albion College, Albion, MI 49224, USA; kkearle@albion.edu

**Keywords:** mass-interpersonal communication, social identification, computer-mediated communication, media enjoyment

## Abstract

Two studies were conducted to test the convergence of mass and interpersonal media processes and their effects on YouTube. The first study examined the influence of interpersonal interactions on video enjoyment. The results indicated that positive comment valence affected participants’ identification with the content creator, which then affected enjoyment of the video. To investigate the effects of convergence from a macro-level perspective, the second study tracked and recorded data from 32 YouTube videos for 34 days and recorded the following data for each video: number of views, likes, and comments/responses. The results indicated that the more content creators and users interact, the more likes the video receives. However, user-to-user interactions are associated with a decrease in the number of likes a video receives.

## 1. Introduction

The literary phrase “breaking the fourth wall” is commonly attributed to French philosopher and art critic Denis Diderot [1]. The term refers to the moment when, often in a dramatic or theatrical presentation, a character addresses an audience directly. While content creators on YouTube may routinely break the fourth wall, the focal interest of this work is a related, but distinctly different phenomenon, the notion of “fifth wall”—a direct two-way interaction between content creators and their audiences. The ”fifth wall” concept challenges the traditional, more passive way of consumption and emphasizes the new paradigm of active engagement between creators of media content and recipients of that content. This unique interaction raises fundamental questions about the impact of “breaking the fifth wall” on content perception and audience enjoyment. This work seeks to address several of the following questions: What happens when a content creator not only breaks the fourth wall but carries on an interaction with an audience member? Does this change the content? Do other audience members enjoy it more? If so, through what mechanism does this occur? In short, what happens when a fifth wall is broken wherein an audience member communicates with a content creator, and what happens when that content creator responds? In other words, how does the breaking of the fifth wall, wherein an audience member interacts with a cast member, affect the ways that people experience media content?

Scholars of computer-mediated communication (CMC) have often approached the study of CMC using two distinctly different lenses. Interpersonal CMC scholars have focused on the ways that relational and emotional nuance is creatively codified into text, how receivers of these messages form impressions, and how those impressions impact various communication functions differently online than they do offline. Alternatively, media scholars who study CMC have explored how message features impact the interpretation of a message, source, and sender based on factors such as credibility or enjoyment. While scholars have illuminated various aspects of CMC from both interpersonal and mass mediated theoretical approaches, more recent work has begun to identify the value of seeking to understand mediated message phenomena from a convergent theoretical perspective that considers both paradigms [2,3,4]. Accordingly, the current study bridges the gap between interpersonal and mass communication theories, offering insights into how one-to-one and many-to-one interactions on platforms like YouTube influence media experiences. In doing so, we aim to expand the scope of CMC research by exploring not just the communication itself but also the subsequent effects on audience identification and content enjoyment, and to contribute to the advancement of theoretical understanding of engagement in the digital age, which is vital as new forms of media continue to emerge and dominate the cultural landscape.

YouTube, a ubiquitous and extremely popular video streaming service [5], is an excellent context for exploring convergent CMC processes and effects as it simultaneously facilitates one-to-many (mass media), one-to-one (interpersonal), and many-to-one communication. Such platforms allow for interpersonal access (whether real or perceived) to the creators of messages intended for mass audiences, potentially enabling users to develop feelings of identification with the authors of media content beyond the content of the media they produce. In and of itself, identification with a content creator may not be entirely novel, but social media, such as YouTube, availing users the opportunity to share messages directly with content creators is. The effect of these interactions on identification with a content creator and the subsequent enjoyment of their content is not yet well understood. 

The present work utilizes YouTube as a venue for exploring theoretically exigent questions surrounding the convergence of mass and interpersonal communication by examining the effects of communication with content creators on observers’ enjoyment of their content in a series of two studies using distinct methodologies. Using experimental methods to manipulate message and communicator characteristics, the first study explores how identification with the content creator affects users’ attitudes toward media content. Functionally, the first study probes what can happen when carefully controlling and manipulating user–creator interactions. The second study further investigates the effects of communication with content creators by analyzing actual behavioral data scraped from a variety of YouTube videos (and their comment sections) each day for 34 days. Together, these studies investigate multiple facets of one intersection between interpersonal and mass communication. 

## 2. Study 1

### Social Identification

Social identity theory [6] sheds light on how individuals perceive themselves and others within the context of group memberships. According to SIT, people categorize themselves and others into social groups based on shared characteristics, such as race, gender, or even more specific affiliations like sports teams or workplace departments. These group memberships constitute a crucial aspect of an individual’s self-concept and exert a profound influence on their attitudes, behaviors, and interactions with others.

Previous research has explored the consequences of this identification process. Individuals who strongly identify with a particular group are more likely to exhibit in-group bias [7] and experience fewer uncertainties [8] compared to those for whom group identification is less salient. This highlights the significant power of in-group identification over our perceptions. In today’s interconnected world, social media platforms play a pivotal role in shaping how individuals form and express their social identities. These platforms provide users with unprecedented access to individuals of international renown, introducing various effects on users’ expectations and their ability to identify with well-known persons [9,10]. Therefore, we hypothesize a similar effect will occur in a social media environment where individuals who have higher identification with the content creator will perceive the content itself more positively.

**H1:**  *Identification with a content creator is positively associated with attitudes toward their content*.

Research in the field of computer-mediated communication (CMC) has frequently examined the role of comments and feedback in online environments. Online comments serve as a prominent avenue for users to engage with content and communicate with content creators. This interaction between the audience and content creators can significantly influence users’ attitudes, behaviors, and their overall identification with the content creator.

Sharing emotional connections, such as positive contact with members, has been shown to foster a sense of community in virtual environments [11]. On one hand, users who encounter a barrage of positive comments while engaging with content on platforms like YouTube are more likely to perceive the content creator favorably and may feel a stronger connection or identification with them. This alignment of positive sentiment in the comment section with the content creator’s message can enhance users’ overall experience and reinforce their identification with the creator [12]. On the other hand, negative comments might encourage users to consider their in-group identification with the content creators. As individuals often seek to enhance their self-esteem by identifying with groups that have a higher social status or greater social desirability [6], we propose that positive comments will have a positive effect on identification with a content creator.

**H2:**  *Identification with a content creator will be significantly higher for those viewing positive comments than for those viewing negative comments*.

Identification has also been suggested as a mediator and/or a moderator rather than an outcome or an effect [8,13,14,15]. The interaction between communication valence and social identification has been shown to influence positive attitudes towards the content itself [16]. Ballouli and colleagues [13] found that the positivity or negativity of an article about a team did not affect participants’ intention to enjoy a game (e.g., buy tickets), even when they exhibited strong identification with the team. However, compared to people who were exposed to a negative message, those who read positive message were more likely to buy tickets to the game at a higher price. Similar effects can be seen in other forms of media. Negative messages written by strong social identification ties have been shown to reduce positive evaluations toward the video content itself [15]. Taken together, identification and message affect likely influence impressions, but the relationship path is somewhat unclear.

While the previous literature has examined identification with the message creator, the present research is interested in the identification with the content creator based upon others’ comments. Therefore, we seek to understand the possible power of identification with the content creator on reducing the effect of comments written by others. To this end, we ask the following research question:

**RQ1:**  Does identification with content creator mediate the relationship between communication valence and attitudes toward the content?

It’s crucial to consider both the identities of commenters and the sentiment of their content. Consumerism research has shown that celebrity endorsements can significantly influence people’s positive perceptions of a brand, e.g., [17]. When comments challenge the prevailing news framing, the high status of commenters becomes a noteworthy factor [18]. Therefore, in such instances, whether a high-status celebrity comments positively or negatively could impact the role of identification as a mediator.

For a more comprehensive understanding of these dynamics, one must also take into account the identity of the respondent. Lee and Shin’s research [19] revealed that when politicians engage with their followers, it fosters a more favorable attitude among people. This aligns with our hypothesis that interaction enhances identification, especially when the respondent is a well-known figure. However, recent research [20] suggests that, rather than a celebrity persona, individuals who are relatable, like another user, may have a more substantial influence on users’ content enjoyment. 

Hence, whether readers observe a response from the content creator or not may affect the significance of the main commenter and the sentiment of the message concerning identification. Social media websites such as YouTube allow users to start their own main comment or respond to another’s comment as a respondent. The interactions between different commenters and the content creator provides a unique setting to examine the effects of identification. Consequently, this study poses the following research question:

**RQ2:**  Does the interaction between the main commenter identity and the respondent’s identity moderate the mediation effect of content creator identification on the relationship between the effect of the message and positive attitudes toward the content?

## 3. Materials and Methods

### 3.1. Research Design Overview

To explore the influence of various interaction types in an online video sharing platform on enjoyment of the video shared, a 2 (main commenter: unknown user vs. Disney) by 3 (secondary commenter: unknown user vs. content creator vs. no commenter) by 2 (comment valence: positive vs. negative) between-subject factorial design experiment was conducted. Across all conditions, an unknown commenter with no reply and the number of likes were held constant. 

### 3.2. Participants and Procedure

A total of 389 participants were recruited from a large university in the Midwestern United States from 3 April to 7 August 2019. After removing data from 15 participants who did not complete the study, 13 people who finished the study in 30 seconds after completing the video, and 20 people who chose the profiles of unknown users as the content creator (i.e., failing a basic attention check), 341 participants were used in analysis. The study was advertised through a university subject pool and participants were granted course credit upon completion of the study. Participants ranged in age from 19 to 37 (*M* = 20.90 and *SD* = 1.74) and most identified as Caucasian/white (69.50%), followed by African American (12.90%), Asian (8.50%), mixed race (3.52%), Latinx (2.35%), Middle Eastern or Arabic (0.88%), other (0.59%), Native Hawaiian or Pacific Islander (0.29%), and American Indian or Alaska Native (0.29%). A total of 4 people reported they preferred not to say. Slightly fewer participants identified as female (43.99%) than male (55.72%). Upon agreeing to participate, participants were informed that they would be shown a YouTube video and comment section. After watching the video and reading through comments, they were asked to answer enjoyment and other self-report questions.

### 3.3. Stimuli Materials

The stimuli were created by using a real YouTube comment section as a template and modifying the names, pictures, and the content in comments temporarily on the website by using Inspect Element feature in a Chrome web browser. This allowed the researchers to maintain the exact style of the webpage, including things like fonts and layout, while changing the information written in the comments. This provided an authentic and realistic stimulus design. After changing the information and picture, researchers acquired screenshots of the artificial comment section to be used as experimental stimuli. In total, 12 comment pages were created for the 2 × 3 × 2 between-subject experiment using this method. The video used in each condition consisted of a parody trailer for the Disney movie, Moana, created by the YouTube user, Screen Junkies. The parody used scenes from the official movie and was paired with a humorous voiceover. Each comment condition included a single comment written by an unknown user. This comment was followed by a brief comment exchange representing each of the 12 experimental conditions. 

The main commenter condition was manipulated by using an unknown user ”Ciara Chung” with a cartoon picture as a profile photo vs. using Disney channel’s official photo and name along with a verified user checkmark. The secondary commenter condition was manipulated by changing the names and pictures of those who replied to the main commenter’s comment. There were three different versions: a main comment with no reply, a reply from an unknown user, or a reply from the content creator, Screen Junkies. Consistent with YouTube’s design template, the Screen Junkies profile had a darker background font indicating they were the content creator. While the name and the pictures varied across conditions, both the unknown user and the content creator always replied “I agree with you 100%” and included a winking emoji.

**Figure 1:**
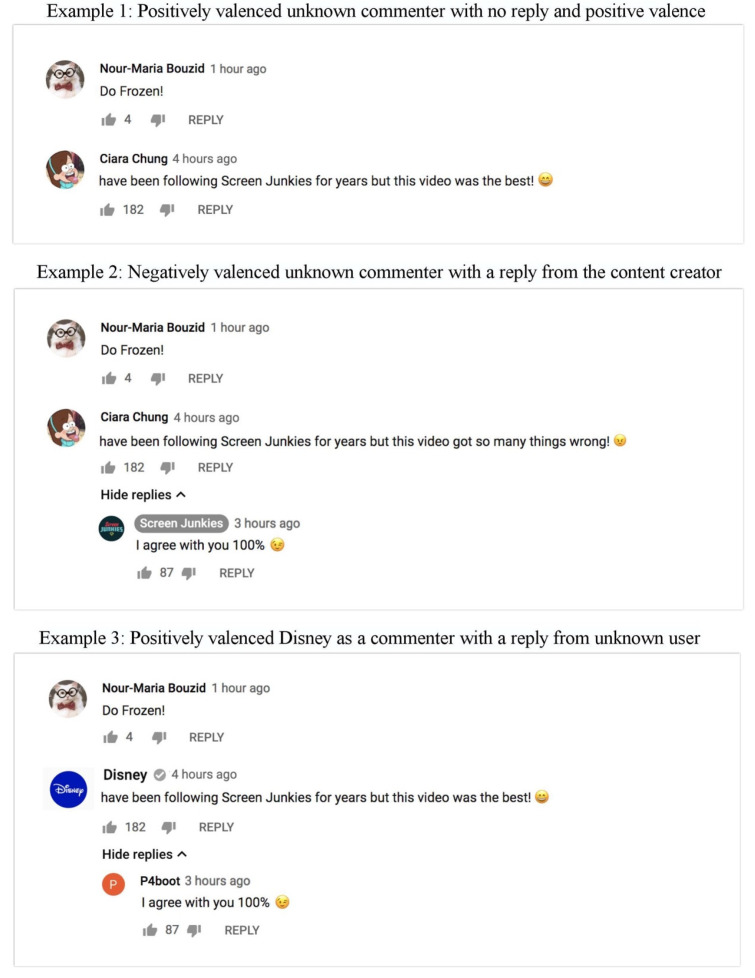
Example conditions for Study 1.

Finally, the content valence condition was manipulated by changing the ending of the comment, “have been following Screen Junkies for years but this video…”. For the positive valence condition, the comment ended with “was the best!” and included a smiling emoji, whereas for the negative valence condition, it ended with “got so many things wrong!” and included an angry emoji (See Figure 1).

### 3.4. Measures

The dependent variable, enjoyment of the video content, was measured using the affective enjoyment sub-scale of Krakowiak’s enjoyment scale [21]. Two items were reverse coded so that higher values indicated a higher level of enjoyment. Items were assessed on a 5-point Likert scale ranging from 1 (strongly disagree) to 5 (strongly agree). The 8-item scale showed an acceptable reliability, *α* = 0.94 (i.e., “I had a good time watching this video” and “It made me happy to watch this video”; *M* = 3.38 and *SD* = 0.97). 

Identification with the content creator was measured using the in-group ties sub-scale of the social identification scale [22]. The original scale was modified for the context of the present study (i.e., instead of “I don’t feel a sense of being ‘connected’ with other (ingroup members)”, “I don’t feel a sense of being ‘connected’ with this user, Screen Junkies” was used.). It was measured on a 5-point scale with endpoints ranging from 1, strongly disagree, to 5, strongly agree. After dropping the reverse-coded items, Cronbach’s alpha score for 6-item scale showed acceptable reliability (α = 0.90; *M* = 2.57 and *SD* = 0.89). 

Before conducting analyses, the data were cleaned of participant cases that did not meet a minimum threshold for attention to the study procedures. To assess this, participants were asked to identify whether the comment valence was positive or negative. There were 97 participants who identified the content valence in a manner that was not consistent with the experimental comment valence condition to which they were assigned. Given that correctly identifying the valence of the main comment on the video would only have required minimal attention to the experimental task, we removed those who failed this manipulation check from further analysis. The resulting final sample contained 244 participants who were used in the hypothesis testing. Although otherwise similar to the original data set, the refined sample included slightly more male (58.20%) than female (41.39%) participants with ages ranging from 19 to 30 (*M* = 20.80 and *SD* = 1.53). Although this procedure generally strengthened observed effect sizes, it had minimal effect on the direction and the observed statistical significance of hypothesis testing results.

## 4. Results

The first hypothesis predicted that identification with a content creator was positively associated with enjoyment of the content. A correlation analysis between identification with the content creator and enjoyment demonstrated that identification was positively related to enjoyment (r = 0.47 and *p* < 0.001). This finding was consistent with H1.

**Figure 2:**
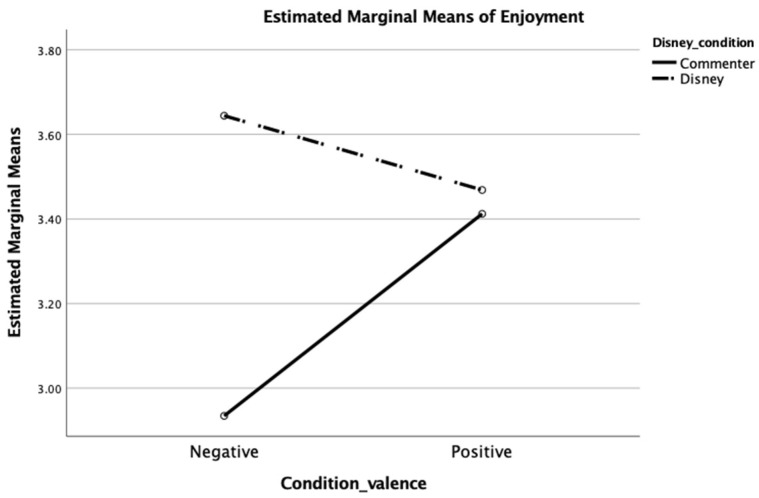
Interaction between message valence and main commenter identity on enjoyment.

The second hypothesis predicted that positive comments would be associated with greater identification with a content creator than when an observer was exposed to negative comments. Participants who observed a positive main comment felt stronger identification with the content creator (M = 2.71 and SD = 0.89) than those who saw a negative main comment (M = 2.35 and SD = 0.88); t (242) = −3.08 and *p* < 0.01, which are consistent with H2. To probe for potential interactions which may have qualified this observed main effect, possible interaction effects on both identification and enjoyment were tested with univariate analyses. One additional main effect was observed. Participants exposed to Disney as the main commenter felt stronger identification (M = 2.71 and SD = 0.91) than when an unknown user was the main commenter (M = 2.44 and SD = 0.87); t (242) = −2.39 and *p* < 0.05. Additionally, an interaction effect between content valence and main commenter on enjoyment was observed; F (1,232) = 6.56, *p* < 0.05, and partial η^2^ = 0.03). Participants who were exposed to Disney as the main commenter enjoyed the video similarly regardless of Disney’s comment being positive or negative. However, those who were exposed to a negative comment written by an unknown user reported less enjoyment than those who were exposed to a positive comment written by an unknown user or any comment written by Disney (see Figure 2). 

The first research question asked whether identification with a content creator mediated the relationship between the comment valence participants observed and their attitudes toward the content. A mediation analysis was run to study if the comment valence (positive and negative) had an indirect effect on enjoyment through identification with the content creator while controlling for gender. Hayes’ PROCESS macro [23] was used to acquire a point estimate of the indirect effect. There was a significant indirect effect, point estimate = 0.19 and 95% CI [0.07, 0.33], suggesting that the effect of comment valence on content enjoyment was partially mediated by identification with a content creator. 

The second research question asked about the potential of interaction effects that may qualify the mediation between comment valence, a content creator’s identity, and the observers’ attitudes toward the content provided. To address this question, we conducted a moderated mediation analysis ([23] Model 11) with main and secondary commenters while controlling for gender. Moderated mediation showed a significant, indirect effect of content valence on enjoyment through identification with the content creator when the main commenter was an unknown user and the secondary commenter was either a content creator, point estimate = 0.32 and 95% CI [0.02, 0.68], or there was no secondary commenter at all, point estimate = 0.33 and 95% CI [0.08, 0.61]. This analysis suggested that the observed mediation from RQ1 was dependent upon the identity or lack of presence of a secondary commenter (Table 1). 

## 5. Discussion

Study 1 evaluated the effects of interpersonal elements (e.g., commenter’s identity and valence of comment content) in a user-generated video sharing platform, YouTube, on the enjoyment of viewed video content. The results illustrate that participants enjoyed videos more when a well-known, public persona (in this case, Disney) wrote a comment in response to video content. Interestingly, the valence of the comment under the video did not affect participants’ reported enjoyment of the video directly on its own. Rather, the valence of the comment affected participants’ identification level with the content creator which, in turn, affected enjoyment. This result extends research demonstrating the effect of the relationship between content valence and social identification on video evaluation [15]. The current research posits that valence of the comment relating to the video content can affect not only identification with the comment writer as studied previously but also identification with the content creator. 

The findings on the observed interaction effect suggest that when a negative comment is written by a peer (unknown user), it affects enjoyment negatively but when it is written by a well-known celebrity user, the effect of valence disappears. One possible reason for that can be that when there is a well-known person or entity commenting under the video, the fact that the celebrity themselves commented under the video may be enough to make it enjoyable. Another reason could be due to identification. Dai and Walther’s research [10] demonstrated that people are more likely to identify with an unknown user rather than a famous person. 

If one were able to experimentally control an online environment, one could ostensibly affect or, at the very least, predict media enjoyment by way of the kinds of user-to-user interactions present in the social media environment. As such, the results of this study help to establish what could happen in a circumstance of high control and help to verify the process of observing an interaction with a content creator leading to identification with that creator which in turn may affect content enjoyment. However, it is difficult to conclude what does happen in a circumstance of low control. In order to explore a less controlled environment and study the effect of social relationships on media content enjoyment, the next study observes the natural environment on YouTube.

## 6. Study 2

A second study explored the effects of content creators and peer interaction on the enjoyment of video content on the video streaming service YouTube. However, because this study capitalized upon publicly available behavioral data rather than participants’ self-reported attitudes, as in Study 1, slightly different hypotheses are presented to reflect publicly available data on a YouTube page. This work proposes that one behavioral indicator of content enjoyment is the “like” function, of which YouTube users may opt to avail themselves. The first prediction suggests that being able to observe interactions between others on a YouTube page affects users’ affect toward a video. Thus:

**H3:**  *An increase in the number of total interactions leads to an increase in daily likes in a video*.

Second, this study sought to explore the ways that observing interactions between a content creator and their audience as well as communication amongst audience members impacted the audience’s enjoyment of the video. To accomplish this, the present study explored two independent variables: creator–commenter interactions and commenter–commenter interactions and evaluated the relationship between these variables and daily likes:

**H4a:**  *An increase in the number of total creator–commenter interactions leads to an increase in daily likes*.

**H4b:**  *An increase in the number of total commenter–commenter interactions leads to an increase in daily likes*.

Lastly, the current study also aspired to investigate the emotional tone of the observed interactions and its effect on audiences’ enjoyment of the video. The last two hypotheses propose that the emotional tone of the interactions is related to the enjoyment of the video content.

**H5a:**  *An increase in the number of total positive interactions leads to an increase in daily likes*.

**H5b:**  *An increase in the number of total negative interactions leads to a decrease in daily likes*.

## 7. Method

### 7.1. Data Collection

The paper mainly focuses on the movie entertainment context. Researchers specifically selected three representative YouTuber entertainment accounts for further analysis: Screen Junkies (6.66 million followers), Cinemasins (8.67 million followers), and WatchMojo.com (accessed on 31 October 2019). (21.30 million followers). In Study 1, researchers selected Screen Junkies as a prototype to create stimuli. To be consistent with Study 1, Study 2 focused on similar YouTubers, Cinemasins and WatchMojo.com (accessed on 31 October 2019). All three YouTubers are the top creators in the movies’ secondary creation discipline that attract sizable followers. We started with Screen Junkies, as they were the content creators used in our initial design, and selected two similar channels based on YouTube’s algorithm-based recommendations and Google searches conducted in March 2019. Researchers traced 32 videos that were published from 17 April 2019 to 2 May 2019. Starting from the date of video publication, we collected data from the main page for each video for 34 days, recording features such as number of views, number of likes, and number of dislikes. Additionally, the comment content was collected verbatim each day. Comments were separated into two categories: comments without replies and comments with replies. The current study defines interaction as both the comment that has a reply and the reply to the comment. The interaction was also divided into two subgroups: (1) interactor identity (creator–commenter and commenter–commenter) and (2) emotional tone (positive and negative). These examined features are described in Table 2 in more detail.

### 7.2. Measurement of Variables

This study necessitated three models. Daily likes was the only dependent variable in the study and was defined as the number of likes a video receives per day. The first model included one independent variable: total interactions. The total interactions included all comments and their replies. For instance, if a single comment was to be followed by four replies, then the total interactions would be calculated by combining the main comment and replies to the comment (1 comment + 4 replies = 5 total interactions). 

The second model included two independent variables: total creator–commenter interactions and total commenter–commenter interactions. Total creator–commenter interactions included the combination of all interactions led by a creator’s comment and all replies from the creator. Other interactions were considered commenter–commenter interactions. For instance, if a creator’s comment was followed by four replies, and the creator also replied to a different comment once, the total creator–commenter interactions would be 1 creator comment + 4 replies + 1 creator reply = 6. 

The third model included two independent variables: total positive interactions and total negative interactions. The total number of positive or negative interactions were defined by the linguistic inquiry and word count (LIWC) software package’s negative or positive emotional word lexicon and analyzed using the LIWC software package (2015) [24], using the equation positive score–negative score. If the overall score was positive, the content would be more positive than negative, and vice versa. The magnitude of this score represented the extremity of the emotional content of the comment. The LIWC dictionary is one of the most popular tools used for sentiment analysis. LIWC reads a given text and compares each word in the text to the list of dictionary words and calculates the percentage of total words in the text that match each of the dictionary categories [24]. For example, if LIWC analyzes a single speech containing 1000 words and finds that 50 of those words are related to positive emotions, then the positive emotion of the speech is 5%. 

In addition to the independent and dependent variables proposed in the hypotheses, the current study necessitated several control variables. Because the number of daily views naturally contributed to the number of daily likes, and the video date (which referred to how long the video had been posted) also influenced the daily likes, daily views and video date were used as level 1 control variables. Because different video content also influenced people’s liking, the video name was coded as a dummy variable, then used as a level 2 control variable. 

### 7.3. Multi-Level Linear Regression Model

**Figure 3:**
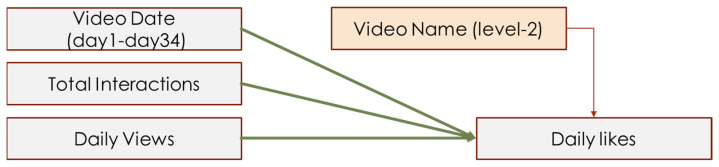
Interaction-like model of H3.

**Figure 4:**
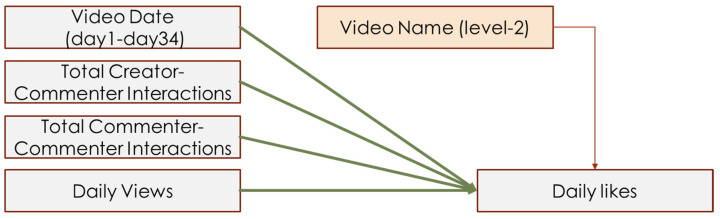
Interaction-like model of H4.

**Figure 5:**
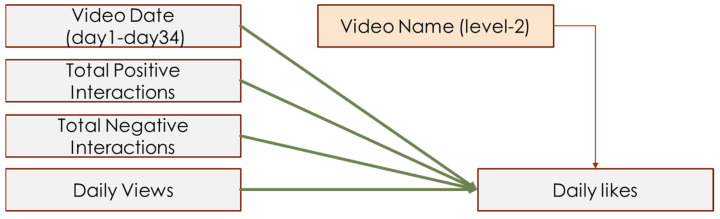
Interaction-like model of H5.

A total of 32 videos were traced for 34 days, recording behavioral observations daily. Therefore, each video similarly contributed to the total daily likes among the 34 days, but differentially contributed to daily likes among 32 videos. Overall, the current study collected 33,768 comments with no replies and analyzed 19,280 interactions (comments with replies). Among all interactions, we have analyzed 1009 creator–commenter interactions, 18,271 commenter–commenter interactions, and analyzed 4522 negative interactions and 5956 positive interactions. Therefore, a multi-level linear regression model (MLM) was used to analyze this data. The current study treated all IVs and control variables as level 1 variables, then specified a dummy variable of video name as the level 2 variable. Different video names had different intercepts of regression, which referred to the video content’s influence on daily likes. Hypothesis models are depicted in Figure 3, Figure 4 and Figure 5. 

## 8. Analysis: Evaluation of MLM Assumptions

To address the question of interest, we tested a multi-level model with different days of videos nested within video names. Paterson and Goldstein [25] recommend at least 25 units per level, on average, for the stability of estimation within an MLM, with full maximum likelihood estimation in every model [26]. In the current MLM model, level 1 (video by day) had 1088 units and level 2 (video name) had 32 units, which was fit for constructing the model. 

The data were explored for the adequacy of sample sizes and missing data, absence of outliers at each level, absence of multicollinearity and singularity, and independence of errors (interclass correlation) [27]. However, level 1 variables, including daily likes, daily views, total interactions, total creator–commenter interactions, total commenter–commenter interactions, total positive interactions, and total negative interactions, were not fit for the assumptions of normality and linearity. Therefore, logarithmic transformations were applied. See Table 3 for descriptive statistics.

### 8.1. Constructing the Unconditional Model

The unconditional model included daily likes as the DV and did not include any independent variables; it is represented by the equation, daily likes = $\beta_{0}$ + r. The intercept was significant at 1.83, t = 28.21, and *p* < 0.001. The intraclass correlation coefficient indicated that 45.12% of the variance in daily likes might be attributed to the difference among videos. These preliminary results of the unconditional MLM warranted that the MLM analysis could further explain the variance of daily likes. Thus, because the unconditional model met the requisite assumptions of the MLM analysis, we proceeded to construct the full model.

### 8.2. Constructing the Full Model

Level 1 variables were centered around their group means and included the following variables: (1) daily views, video date, and total interactions for the H3 model; (2) daily views, video date, total creator–commenter interactions, and total commenter–commenter interactions for the H4 model; and (3) daily views, video date, total positive interactions, and total negative interactions for the H5 model. The level 2 variable only influenced the level 1 intercept ($\beta_{0}$), so the full model was developed by adding level 1 coefficients to the unconditional model. The full model for H3 was represented by the following equations. In the H3 model, the intercept was significant at 1.83, t = 12.73, and *p* < 0.001. Comparing the deviance from the unconditional model (−595.80 and df = 3) and the full model for H3 (410.45 and df = 6), the full model for H3 was superior, Δχ^2 (3) = 2012.5 and *p* < 0.001, accounting for 40.26% of the variance in level 1. In the H4 model, the intercept was significant at 1.83, t = 14.43, and *p* < 0.001. Upon comparing the deviance from the unconditional model (−595.8 and df = 3) and the full model for H4 (431.23 and df = 7), the full model for H4 was superior, Δχ^2 (4) = 2054.1 and *p* < 0.001, accounting for 43.58% of the variance in level 1. In the H5 model, the intercept was significant at 3.795, t = 10.471, and *p* < 0.001. Upon comparing the deviance from the unconditional model (−595.8 and df = 3) and the full model for H5 (421.47 and df = 7), the full model for H5 was superior, Δχ^2 (4) = 2034.5 and *p* < 0.001, accounting for 42.96% of the variance in level 1):

**Equation for H3:**  Daily likes = $\beta_{0}\;$+ $\beta_{1}*daily\;view\;$+ $\beta_{2}*\;video\;date\;$+ $\beta_{3}*\;total\;interaction$ + r

**Equation for H4:**  Daily likes = $\beta_{0}\;$+ $\beta_{1}*daily\;view\;$ + $\beta_{2}*\;video\;date\;$+$\;\beta_{3}*total\;creator-commenter\;interaction$ + $\beta_{4}*total\;commenter-commenter\;interaction$ + r

**Equation for H5:**  Daily likes = $\beta_{0}\;$+ $\beta_{1}*daily\;view\;$+ $\beta_{2}*\;video\;date\;$+ $\beta_{3}*total\;positive\;interaction$ + $\beta_{4}*total\;negative\;interaction$ + r

## 9. Results

According to the results in Table 4, this study concluded that both daily views and video date positively contributed to daily likes across the three models (all *p* values < 0.001). The intercept, which was the level 2 control variable (video name), significantly helped explain all three models (all *p* values < 0.001), meaning that the specific content of a video evoked different levels of initial liking of specific videos. 

Hypothesis three predicted that the overall number of interactions on a video would be significantly related to the amount of likes a video received. The results demonstrate that total interactions negatively influenced daily likes (β = −2.12 and *p* < 0.001). That is, the more total interactions a video had, the fewer daily likes the video received from users. Therefore, the data were not consistent with H3. 

The current study also looked at the interactions among other users with one another and with content creators and predicted positive relationships between both user-to-user interactions and user-to-content creator interactions and liking of specific videos. Total content creator–user interactions positively predicted daily likes (β = 0.34 and *p* < 0.001), while total user-to-user interactions negatively predicted daily likes (β = −2.54 and *p* < 0.001). That is, if the video creator commented, the number of likes increased. Interactions among other users did not increase the number of likes on the video. The data were consistent with H4a, but not with H4b. 

The total negative interactions positively influenced daily likes (β = 0.61 and *p* < 0.005), and total positive interactions negatively influenced daily likes (β = −2.47 and *p* < 0.001). These findings were in the opposite direction to what was predicted. Thus, the data were not consistent with hypothesis 5a or 5b. 

## 10. Discussion

Study 2 examined the effects of different types of interactions on liking behavior. The results indicated that videos upon which a content creator interacted with other users in the comment section increased liking more than videos without content creator interactions. The first model looking at the effect of the total interactions on liking behavior showed a negative effect. The reason for the total influence of interactions having a negative effect on the number of likes in the first model may be due to the driving force of the user-to-user interactions, which constitute a large portion of the interactions under the video. 

Furthermore, the current study finds that negative comments lead to a greater number of likes whereas positive comments lead to a smaller number of likes. A possible explanation for this could be increased motivation for content supporters to mobilize. Basically, negative interactions might encourage supporters of the content to react positively (e.g., liking the video) in defense or support. Correspondingly, such a relationship would also explain why positive comments might produce fewer positive content interactions as the positive comments may elicit a weaker emotional response to the content. Therefore, participants might have agreed with, or even enjoy, the content but did not feel a strong enough compulsion to actively show support (e.g., like). A similar yet different explanation could be the contrast effect. In other words, viewers might like the content to counteract the negativity in the comments, thus, increasing the number of likes. On the other hand, if a video is already well-received and supported by positive comments, additional likes may be seen by viewers as unnecessary. Lastly, another possible explanation could be that a positive (or a negative) interaction did not necessarily mean a positive (or a negative) attitude towards the video itself. For instance, a negative interaction defending criticism to a video could be considered as a favorable sentiment toward the video itself. However, the current analyses only looked at the overall emotional tone of each comment and not the meaning of a comment in a specific context. Future research might explore these aspects in greater detail to unravel the nuanced dynamics of social media engagement. We strongly encourage others to dive deeper into the dynamic processes of emotional engagement and reaction in social media and to investigate contrast effects to determine if individuals are more likely to show support to a greater degree if content they like receives negative comments. Lastly, future research should consider the possibility that a large portion of the comments that are attached to YouTube videos do not centrally concern the content of a video itself. Moreover, it may be the case that one of the strongest behavioral indicators of media content enjoyment may be the presence of individuals who defend that content against detractors—or that the presence of detractors provides a defensive function which, perhaps according to a mechanism of psychological reactance [28], leads some content to be perceived more positively than that content would otherwise would have been perceived. 

Future work should evaluate whether those users, who may have experienced an adverse reaction to detractors, experienced this reaction in part due to their identification with particular content creators. If one feels that one has developed a relationship with a content creator—even if just through YouTube—one may also feel it is their duty to defend said content creator.

## 11. Overall Discussion 

The results from the behavioral data collected in Study 2 were generally consistent with that of the lab experiment conducted in Study 1. Both the first and the second study indicated that interpersonal messages contribute to mass communication effects. For instance, comment interactions under the video content influenced people’s enjoyment of the video. While the uncontrolled nature of the second study cannot provide individually distinct observations such as trait and perception differences across participants, based on these two studies, the effect of observing communication between a content creator and other users on an affective reaction or behavioral response to a video provides evidence that the displayed relationships between content providers and their audience may well affect an audience’s enjoyment of content. 

Overall, these studies have attempted to examine a user-generated video-sharing social platform from a convergent theoretical perspective suggested in previous research [3,4,29]. Results of the first study and the observations from the second study demonstrate that mass media and interpersonal effects are likely happening concurrently on YouTube. Not only does media content affect an individual’s positive disposition and intentional behaviors but, also, interpersonal communication happening under the video affects the perception of the video content. 

Both studies sought to understand the ways social identification with a content provider may affect the enjoyment of media content. In Study 1, participants’ identification with a content creator facilitated respondents’ enjoyment of the media content itself. However, in the behavioral data analysis, observing more content creator interactions increased the number of likes to the video per day. Data collection methods also helped elucidate these processes. The experiment presented in Study 1 provided a carefully controlled examination of the effects of a specific content-creator-written message on participants’ reported attitudes toward the media content itself. The observational work in Study 2 provided a large-scale observation of a series of videos while recording users’ interactions with those videos. This allowed for the collection of a reasonably large amount uncontrolled field data reflecting users’ commenting behavior and the interactions with the media content itself. 

The results from Study 1, which demonstrate that the effect of comment valence on media content enjoyment is facilitated by social identification with the media content provider, provide one plausible explanation for the discrepancies in the influence seen in Study 2. Users appear to develop feelings of identification with the authors of media content not only because of the content of the media they produce, but also because of the messages they directly share with their viewers and even those messages the viewers share amongst themselves. The process of social identification with content providers should be examined, in greater granularity, using the specific qualities of the communication generated by content creators and how those messages affect individuals’ social identification with those creators.

This study also raises questions about the effect of content valence. While content valence contributes to people’s enjoyment, it only does so in certain situations. For instance, the first study partially supported the effects found in previous research which showed the impact of negative comments on people’s perception and attitude towards the content, e.g., [30]. Nonetheless, the second study indicates that a wider variety of situations should be taken into consideration. For example, examining content valence when commenters provide negative messages to defend the video, or the content valence when commenters provided positive messages to other commenters’ negative messages. Whether these situations should be defined as simple valence categories, positive or negative, and how they contribute to the enjoyment of videos is one potential avenue for future research.

While the second study focused only on the content creator as a sole, well-known user, the results from the first study have shown the influential effect of a secondary well-known user who is not the content creator on audience responses. Once we controlled for the secondary well-known commenter, content valence no longer affected participants’ reported enjoyment of the video content. These results contradict previous literature demonstrating the effect of negative comments by a well-known user on Twitter on future behavioral intention and involvement [14]. However, their research did not look at the effect of interpersonal communication. This again stresses the importance of studying the new social media environment through convergence perspectives which consider both mass media effects and interpersonal processes. 

### Limitations and Future Directions

Notwithstanding their contributions, these studies are not without their limitations. First, despite results being mostly replicated in Study 2, the reliance on a student population from a single large university in the Midwestern United States might limit the extrapolation of findings to other demographics or geographic locations. 

Furthermore, we observed 97 participants who were unable to evaluate condition valence correctly (and hence, excluded from analyses) in Study 1. Our findings align with previous research, which has shown predicting the valence of an online comment perfectly is a nearly impossible task to accomplish, e.g., [31,32]. Future research could investigate the factors influencing misinterpretation, such as individual psychological traits, cultural backgrounds, and the specific context of the comments. Additionally, exploring the role of non-verbal cues, or their absence, in digital communication platforms could provide valuable insights. This line of inquiry could lead to the development of more nuanced methodologies for assessing valence in online interactions.

Also, the second study collected behavioral data on YouTube once per day, which may have provided too long of a time interval between observations. Because of this relatively long lag in the data collection interval, this study may have missed some fairly important moments in the development of the media content. There may have been smaller increments of time in which key communicative acts occurred and meaningfully affected our outcome variables, which were lumped together into the day’s aggregate data. It is important to consider that data can be compressed easily, but once these data are collected it is difficult, if not impossible, to opt for a more granular approach. 

As noted above in the discussion of Study 2, future research ought to improve the ways that creator-to-consumer and user-to-user interactions are analyzed. The present work utilized LIWC as a tool to generally assess the nature of the linguistic content observed in the comment sections of videos included in Study 2. While LIWC has a number of demonstrable strengths, utilizing other tools (i.e., utilizing advances in AI, machine learning, and natural language processing approaches) to better understand the comments generated on videos within their appropriate context may provide much clearer answers about why, precisely, some of the effects we observed emerged. We believe there is a great deal more clarity to be gleaned from future work utilizing such approaches.

Lastly, the second study coded all replies written to a content creator’s comment as content creator–commenter interaction. Future researchers may also want to look at secondary level content creator–commenter interaction by looking at the replies that receive a re-reply from the content creator. Given the strong effects that interaction with a content creator has on an audience’s enjoyment and feelings of identification with that content creator, we hope future research will help to better explain the effect of observation of content creators’ communication. Also, it is quite possible that the order of a content creator’s comments and users’ comments is quite important in determining the degree of social identification observers infer from observing these interactions. It seems plausible that when a content creator replies to a user there may be a different identification effect observed than when a user replies to a content creator. After all, anyone can reply to a message from a content creator, and the content creator may have seen that message or not—the degree of interaction with that individual is uncertain. However, when a content creator replies to another user, it is no longer plausible that they have not seen and considered that user’s message. Perhaps some types of users and content creator interaction are more effective at affecting users’ perceptions of identification with a content creator.

## 12. Conclusions 

This research investigated a mechanism of enjoyment from observing interpersonal communication among well-known and unknown users. The findings contribute to the literature on enjoyment and social identification from a convergent theoretical perspective, as well as highlighting potential directions to better grasp the effect of interactions through message source and content valence. This work demonstrates the utility of using diverse methodological approaches to understand the convergence of mass and interpersonal communications present on many social media platforms. It is our hope that this type of approach can provide an attractive and theoretically utilitarian approach to generating and responding to new research questions.

The notion of breaking the fifth wall, as described here, suggests that being able to observe one-to-one interactions between message content creators and users actively affects the ways that mediated message recipients perceive those one-to-many messages that are created and sent by a content creator. Research that lives at the intersection of interpersonal and mass communication has been around for some time, e.g., [33]. This work empirically contributes to this approach. It proposes that much media content, once only a one-to-many deliverable, now, via CMC, can be consumed and discussed alongside interpersonal interaction that occurs among viewers and, importantly, between viewers and media content providers themselves. These interpersonal interactions are recorded and displayed in perpetuity. And, importantly, when viewers are able to break the fifth wall and interact with content creators and providers, it can meaningfully change the media experience for all who observe that interaction.

## Figures and Tables

**Table 1:** Moderated Mediation Analysis.

	Main Commenter: Unknown			Main Commenter: Disney		
	Secondary Commenter			Secondary Commenter		
	None	CC	Unknown	None	CC	Unknown
Enjoyment						
Unstandardized B (SE)	0.33 (0.13)	0.32 (.17)	0.17 (.14)	0.04 (.16)	−0.01 (.14)	0.26 (0.16)
Lower 95% CI	0.08	0.02	−0.07	−0.28	−0.27	−0.06
Upper 95% CI	0.61	0.68	0.50	0.36	0.29	0.57
Conditional mediation	*	*				

Notes. Post hoc results. A total of 5000 bootstrap samples, 95% CI, and Model 11. Asterisk indicates a significant indirect effect and *p* < 0.05. CI = confidence interval and CC = content creator.

**Table 2:** Features of Data Collection.

Feature Level	Feature	Sub-Feature	2nd Sub-Feature
Video level	Video date		
	View number		
	Like number		
	Dislike number		
Comment level	Comment content	Comment without replies	
		Comment with replies, including reply (Interaction)	Positive interactions and negative interactions
			Creator–commenter interactions and commenter–commenter interactions

**Table 3:** Descriptive Statistics of Variables.

	Mean	Centered Mean	S.D.	Skewness	Kurtosis
Video Name	16.50		0.89	0.00	−1.20
Video Date	17.50		0.54	0.00	−1.20
Log daily views	3.26	0.00	0.39	0.08	−0.47
Log daily likes	1.83	0.00	0.84	1.42	1.98
Log interactions	2.55	0.00	0.38	0.21	0.38
Log creator	0.59	0.00	0.48	1.01	−0.49
Log commenter	2.53	0.00	0.39	0.32	0.60
Log negative	1.84	0.00	0.89	−0.10	0.68
Log positive	2.04	0.00	0.54	0.27	−0.28

Note. N = 1088 (listwise). Log creator = log of total creator–commenter interactions. Log commenter = log of total commenter–commenter interactions.

**Table 4:** Multilevel Regression Estimates of Daily Likes.

Variables	Unconditional Model		Hypothesis 3 Model		Hypothesis 4 Model		Hypothesis 5 Model	
	Estimate	*p*	Estimate	*p*	Estimate	*p*	Estimate	*p*
Intercept	1.83	<0.001	1.83	<0.001	1.83	<0.001	1.83	<0.001
Daily views			0.56	<0.001	0.55	<0.001	0.57	<0.001
Video date			0.004	<0.001	0.004	<0.001	0.004	<0.001
Total interactions			−2.12	<0.001				
Total creator–commenter interactions					0.34	<0.001		
Total commenter–commenter interactions					−2.54	<0.001		
Total positive interactions							−2.47	<0.001
Total negative interactions							0.61	<0.005
AIC of the model	1197.6		−808.9		−828.9		−848.5	
BIC of the model	1212.6		−779.0		−794.0		−813.5	
Deviance of the model	1191.6		−820.9		−842.9		−862.5	
Heterogeneity level of the model (ICC)	0.451		0.578		0.558		0.541	

## Data Availability

Data will be made available upon request. Please reach out to corresponding author, Ezgi Ulusoy (ulusoyez@msu.edu).
